# Divergent effects of single and combined stress of drought and salinity on the physiological traits and soil properties of *Platycladus orientalis* saplings

**DOI:** 10.3389/fpls.2024.1351438

**Published:** 2024-06-06

**Authors:** Shan Li, Sen Lu, Jing Wang, Zepeng Liu, Chuhuan Yuan, Min Wang, Junkang Guo

**Affiliations:** Department of Environmental Science and Ecology, School of Environmental Science and Engineering, Shaanxi University of Science and Technology, Xi’an, China

**Keywords:** salt, drought, xylem hydraulics, physiological traits, soil properties

## Abstract

Drought and salinity are two abiotic stresses that affect plant productivity. We exposed 2-year-old *Platycladus orientalis* saplings to single and combined stress of drought and salinity. Subsequently, the responses of physiological traits and soil properties were investigated. Biochemical traits such as leaf and root phytohormone content significantly increased under most stress conditions. Single drought stress resulted in significantly decreased nonstructural carbohydrate (NSC) content in stems and roots, while single salt stress and combined stress resulted in diverse response of NSC content. Xylem water potential of *P. orientalis* decreased significantly under both single drought and single salt stress, as well as the combined stress. Under the combined stress of drought and severe salt, xylem hydraulic conductivity significantly decreased while NSC content was unaffected, demonstrating that the risk of xylem hydraulic failure may be greater than carbon starvation. The tracheid lumen diameter and the tracheid double wall thickness of root and stem xylem was hardly affected by any stress, except for the stem tracheid lumen diameter, which was significantly increased under the combined stress. Soil ammonium nitrogen, nitrate nitrogen and available potassium content was only significantly affected by single salt stress, while soil available phosphorus content was not affected by any stress. Single drought stress had a stronger effect on the alpha diversity of rhizobacteria communities, and single salt stress had a stronger effect on soil nutrient availability, while combined stress showed relatively limited effect on these soil properties. Regarding physiological traits, responses of *P. orientalis* saplings under single and combined stress of drought and salt were diverse, and effects of combined stress could not be directly extrapolated from any single stress. Compared to single stress, the effect of combined stress on phytohormone content and hydraulic traits was negative to *P. orientalis* saplings, while the combined stress offset the negative effects of single drought stress on NSC content. Our study provided more comprehensive information on the response of the physiological traits and soil properties of *P. orientalis* saplings under single and combined stress of drought and salt, which would be helpful to understand the adapting mechanism of woody plants to abiotic stress.

## 1 Introduction

Forests provide great economic and ecological value for human beings, however, are facing a severe threat of the increasing impacts of climate change. Drought conditions, exacerbated by climate change, are expected to become more frequent and intense, leading to large-scale tree mortality (Bradford et al., 2022). Meanwhile, approximately 1 billion hectares of land worldwide are affected by salinity, particularly in arid and semi-arid regions, posing additional challenges to plant survival (Wicke et al., 2011; Hopmans et al., 2021). Woody plants often face the dual stress of drought and salinity in natural conditions (Liu et al., 2022a). Water deficit results in reduced xylem water potential, prohibited xylem water transport and carbon metabolism processes, and ultimately tree death (Cui et al., 2019; Mantova et al., 2022). Similarly, soil salinity inhibits water absorption due to osmotic stress and can lead to ion toxicity, further affecting plant health (Lambers et al., 2008).

In response to drought and salt stress, woody plants show instant biochemical adjustment (Li et al., 2023), such as hormone regulation, e.g., abscisic acid (ABA), gibberellic acid (GA_3_), jasmonic acid (JA) and its methyl ester methyl jasmonate (JA-Me), and indole acetic acid (IAA) (Ryu and Cho, 2015). For example, ABA induces leaf stomatal closure to reduce water loss by transpiration and thus maintains higher xylem water potential under drought or salt stress (Martin-StPaul et al., 2017; Ouyang et al., 2017). GAs play an important role in woody plants’ resistance to salt stress (Colebrook et al., 2014), and their content changes under salt stress may be species-specific, e.g., reduced content was found in *Oryza sativa*, *Phaseolus acutifolius*, whereas increased content was found in *P. vulgaris* (Yurekli et al., 2004; Farooq et al., 2022). Similarly, under drought and salt stress, JA and JA-Me also have a positive effect on stress tolerance (Kang et al., 2008; Daszkowska-Golec and Szarejko, 2013), and the changes in its content were also species-specific (Kang et al., 2005; de Ollas et al., 2013). IAA regulates soluble sugar content, promotes root development and maintains chlorophyll content, which plays a crucial role in plant growth, development, and health (Talebzadeh and Valeo, 2022; Verma et al., 2022). Under drought and salt stress, biochemical traits such as leaf chlorophyll content may increase, decrease, or remain unchanged, depending on the species, and the duration and intensity of the stress (Anjum et al., 2011; Acosta-Motos et al., 2017). Non-structural carbohydrates (NSC), including soluble sugars and starch, represent the balance between carbon supply and carbon assimilation in woody plants, and it has been shown that both drought and salt stress can lead to a decrease in NSC content in woody plants (Sevanto et al., 2014; Lima et al., 2021). NSC content predicts the process of carbon starvation, which occurs when a plant is unable to supply enough carbohydrates for the metabolic processes necessary for survival, leading to plant mortality (Weber et al., 2019; McDowell et al., 2022).

Xylem is the essential tissue responsible for long-distance water transport in woody plants, besides the above-mentioned biochemical traits, the response of xylem hydraulic and anatomical traits under drought and salt stress has been investigated intensively over the last few decades (Polle and Chen, 2015; Lens et al., 2022). Specifically, woody plants could adjust their xylem anatomical traits to balance xylem hydraulic efficiency and safety, hence to avoid the potential risk on its growth and development under drought and salt stress (Liu et al., 2022b; Li et al., 2024). For instance, woody plants construct narrower xylem conduits with thicker conduit cell walls under drought stress (Li et al., 2022b), which show lower hydraulic efficiency but higher safety (Cai and Tyree, 2010; Hacke et al., 2017). Under salt stress, woody plants may also show xylem hydraulic and anatomical changes. For instance, under salt stress, poplar shows decreased vessel lumen and increased vessel wall thickness, although the xylem hydraulic conductivity of salt-tolerant poplar was unchanged, whereas that of salt-sensitive poplar decreased (Junghans et al., 2006).

Drought and salt stress negatively affect soil properties, plant growth and productivity (Ali et al., 2017). Soil microbial communities and nutrient availability, both crucial for maintaining soil fertility, exhibit dynamic responses to environmental stresses, with consequential effects on plant growth (Dai et al., 2019; Xu et al., 2020). The rhizobacterial communities are sensitive to changes in soil moisture and salinity (Rath et al., 2019), with drought conditions known to induce shifts in their composition and function (Naylor and Coleman-Derr, 2018). Salinity stress can also alter microbial diversity and abundance, which is evaluated by alpha diversity (Willis, 2019), and further affecting plant growth indirectly (Ma et al., 2020). Meanwhile, both drought and salt can affect soil nutrient content and effectiveness in ways that alter nutrient uptake by plants and microbes, or the cycling of nitrogen, phosphorus, and potassium (e.g., by affecting nitrogen mineralization, soil phosphorus adsorption-desorption behavior, and mobility of mineral elements) (Chang, 2023).

Previous work has been done on investigating the effects of single stress such as drought and salt on soil physicochemical properties, microbial communities, and physiological traits of woody plants (Daliakopoulos et al., 2016; Li et al., 2023), however, studies on combined drought and salt stresses are still rare. Combined stress may result in very similar or completely different response of plant physiological traits, compared with single stress (Suzuki et al., 2014). Due to the frequent tree mortality events under combined abiotic stress (Niinemets, 2010; Teshome et al., 2020), it is crucial to understand the response of plant physiological traits and soil properties under combined stress, as well as its difference compared with single stress. *P. orientalis* is widely used for ecological restoration in western China and is a major silvicultural species, however, its response to drought, salt, and their combined stress remains unclear. In this study, we aim to investigate the effects of drought, salinity, and their combined stress on the physiological traits of *P. orientalis*, as well as its rhizobacterial communities and soil nutrient (N, P, K) content. We hypothesize that more significant responses would be observed under combined stress compared with single stress, and drought and salt stress may have different effects on different traits. Our detailed investigation on the responses of physiological traits and soil properties under drought and salt stress would be helpful to understand the adaptive mechanisms of this species with abiotic stress.

## 2 Materials and methods

### 2.1 Plant material and experimental design

The experiment was performed in a growth chamber with a light period of 16 h at day/8 h at night, a temperature of 26 °C at day/20 °C at night, and a relative humidity of 50-60%. We determined two salt stress levels by preliminary experiments (150 mmol NaCl, moderate salinity, and 300 mmol NaCl, severe salinity, per kg of soil) and set up two watering patterns (well-watered and slight drought, which corresponded to the soil water concentration of 70-80% of the maximum water-holding capacity of the soil, and the soil water concentration of 40-50% of the maximum water-holding capacity of the soil, respectively).

In April 2023, thirty *P. orientalis* saplings (one per pot) were planted using approximately 2 L pots. All saplings were two years old and about 30 cm tall. The soil used was commercial nutrient soil containing mainly peat, coir and perlite, and each pot was filled with 500 g of soil. After micro-acclimation for one month, the saplings were treated with drought and/or salt stress. Twenty saplings were irrigated with a quantity of 600 mM solution of NaCl to reach the set soil salinity. Subsequently, irrigation was stopped for half of the saplings in each soil salinity (0, 150, 300 mmol/kg) until the soil water content was reduced to 40-50% of the maximum soil water content, which took 15 days. Therefore, there were in total six groups with five saplings in each group, namely control (well-watered, W), well-watered + moderate salinity (WMS), well-watered + severe salinity (WSS), drought (D), drought + moderate salinity (DMS), and drought + severe salinity (DSS). Eventually, the single drought stress, the single salt stress, and the combined drought and salt stress lasted for 30, 45, and 30 days, respectively. The stresses started from May 15^th^, 2023, and ended on July 1^st^, 2023. In each group, 3-5 saplings were used for determination of biochemical traits, xylem hydraulic and anatomical traits, with at least two technical replications included in each sapling for determination of biochemical traits and xylem hydraulic traits.

At the end of the stress experiment, the hydraulic conductivity of the xylem was first measured. Subsequently, we cut each sapling, separating the remaining roots, stems, and leaves for biochemical and anatomical measurements. All sampled leaves were in a mature state at the time of collection, and the leaves used for biochemical index determination were not differentiated by the age.

### 2.2 Determination of plant hormone content

Fine roots and leaves of 3-5 saplings per group were collected for determination of phytohormone content (ABA, GA_3_, JA-Me, IAA) using enzyme linked immunosorbent assay (ELISA) as described by Yang et al. (2001). Briefly, 0.5 g of fresh plant material was weighed, snap-frozen in liquid nitrogen, and ground to a powder, which was subsequently extracted with cold 80% (v/v) methanol with butylated hydroxytoluene at 4°C overnight. After centrifugation and extraction of the supernatant, the extracts were passed through a C18 Sep-Pak cartridge (Waters, Milford, MA), dried in N_2_, and dilution with PBS (phosphate buffered saline). Subsequently, 50 μL of standard samples and diluted samples to be tested were added to a 96-well enzyme plate, followed by 50 μL of diluted samples containing a certain amount of antibody. After incubation at 37°C for 0.5 h, the enzyme plate was subsequently washed with a washing solution containing PBS and Tween-20. The appropriate amount of enzyme secondary antibody was added to the diluted sample, and 100 μL was added to each well of the enzyme plate and incubated at 37°C for 0.5 h, and then the plate was washed again. After washing the plates again with washing solution, o-phenylenediamine was added to develop the color, followed by termination of the reaction with sulfuric acid and measurement of the absorbance values of individual samples at 490 nm.

### 2.3 Determination of leaf chlorophyll content

To determine leaf chlorophyll content, 0.1 g of fresh leaves were collected from each sapling and extracted in 95% ethanol for 24 h. The chlorophyll extract was centrifuged at 8000 r/min for 10 min and the supernatant was taken. The absorbance of the extract was measured at 665 nm, 649 nm and 470 nm, and the chlorophyll A, chlorophyll B and carotenoid contents were calculated according to Equations 1–3 (Zhao and Cang, 2016).


(1) $${\text{C}}_{\text{a}}=13.95\times {\text{A}}_{665}-6.88\times {\text{A}}_{649}$$

(2) $${\text{C}}_{\text{b}}=24.96\times {\text{A}}_{649}-7.32\times {\text{A}}_{665}$$

(3) $${\text{C}}_{\text{c}}=\frac{1000\times {\text{A}}_{470}-2.05\times {\text{C}}_{\text{a}}-114.8\times {\text{C}}_{\text{b}}}{245}$$

Where *C*_a_ is the content of the chlorophyll A, *C*_b_ is the content of the chlorophyll B, *C*_c_ is the content of carotenoid, *A*_665_ is the absorbance at the wavelength of 665 nm, *A*_649_ is the absorbance at wavelength of 649 nm, and *A*_470_ is the absorbance at wavelength of 470 nm.

### 2.4 Determination of non-structural carbohydrates content

NSC content was determined as described in Li et al. (2008). Leaves, stems, and roots of 3-5 saplings per group were collected and immediately dried in an oven at 105°C for 30 min to terminate the enzyme activity, followed by drying at 65°C for 48 h to constant weight. To determine the soluble sugar content, 0.1 g of dried sample from each sapling was weighed and added to 10 mL of 80% ethanol solution, then centrifuged and the supernatant was extracted in a water bath at 80°C for 30 min. To extract starch, the residue was mixed with 10 mL of distilled water and after boiling water bath for 30min, 2 mL of 9.2 M perchloric acid was added, and the supernatant was extracted by centrifugation, and the extraction was repeated for a total of three times. 2 mL of the diluted extract was taken in a test tube, followed by the sequential addition of 0.5 mL of anthrone-ethyl acetate solution (20 g/L) and 5 mL of sulfuric acid (18.4 mol/L). The test tubes were cooled in cold water after a water bath at 100°C for 10 min and the absorbance at 630 nm was determined.

### 2.5 Determination of xylem anatomical traits

To prepare microscopic sections, a 2 cm long sample was selected from the middle of the main stem of 3-5 saplings per group. These samples were then stored in 70% alcohol and later rehydrated in pure water for 1.0 hours. After the rehydration process, the samples were slightly dried at room temperature and fixed on a microtome (Leica RM2126RT, Germany). Sections with a thickness of 20μm were sliced and afterward stained with 1% safranin. After staining for 2 min, the sections were rinsed with pure water, and then dehydrated into 50%, 75%, 95%, and 100% ethanol for several seconds (Li et al., 2022b). For the observation and collection of microscopic images, a light microscope (Olympus CX43, Japan) was utilized. Under 200× magnification, a pie-shaped region of the section with at least 200 consecutive tracheids included was selected from the outermost annual rings of the xylem, and the tracheid double wall thickness and tracheid lumen diameter were measured by the software Image J. For each image, the measurement of the diameter of the lumen and the double wall thickness of the tracheid was selected from the outermost 5 layers of xylem, which were inferred as newly developed xylem during the stress period, due to the great overlap between the xylem growing period of this species and the stress period of our experiment.

### 2.6 Determination of xylem hydraulic traits

Every three days, the midday leaf water potential of each sapling was monitored using a pressure chamber (PMS Instruments, Albany, OR, USA). The leaves for xylem water potential measurements were enclosed in plastic bags that were surrounded by aluminum foil to stop transpiration and balance the xylem water potential of stems and leaves (Begg and Turner, 1970). For xylem hydraulic conductivity measurement, a 2 cm long stem segment was collected underwater from the middle part of 3-5 saplings per group (McCulloh et al., 2014; Maruta et al., 2022), and the xylem specific hydraulic conductivity (*K*_h_) was measured by using the Sperry apparatus and calculated according to Equation 4, where the pressure drop of degassed KCl solution (0.01 mol/L) generates a certain water flow in the stem (Sperry et al., 1988).


(4) $$K_{\text{h}}=\frac{\text{F}\times L}{\text{Δ}P\times A}$$

Where *F* is the velocity of water flow through the stem, *L* (m) is the length of the stem, Δ*P* (MPa) is the pressure difference generating water flow in the stem segment, and *A* is the xylem area of the cross-section of the stem segment. The influence of resin on the determination of xylem hydraulic conductivity was excluded because there were no resin canals in the xylem of *P. orientalis*, and no influence of resin on the determination of xylem hydraulic conductivity was found in the preliminary experiments.

### 2.7 Determination of soil nutrient (N, P, K) content

Non-rhizosphere soil samples used for soil nutrient content were collected and determined with reference to NY/T 1121-2006 soil testing standards (MOA, 2006). Briefly, the ammonium nitrogen (NH_4_-N) content was determined by the potassium chloride extraction-indigo phenol blue colorimetric method; the nitrate nitrogen (NO_3_-N) content was determined by the phenol disulfonic acid colorimetric method. Available phosphorus (AP) content was determined by sodium bicarbonate extraction-molybdenum antimony colorimetry, and available potassium (AK) content was determined by flame spectrophotometry.

### 2.8 Structure of rhizobacterial community

Soil DNA was extracted from 0.5 g low-temperature lyophilized rhizosphere soil of 3 saplings according to the user manual of the FastDNA™ SPIN Kit for Soil (MP Biomedicals LLC, Santa Ana, CA). The concentration and quality of the extracted DNA were determined by NanoDropOne (NanoDrop Technologies, Wilmington, DE, USA). Qualified DNA samples were stored at −80 ^°C^C for subsequent experiments. For bacteria, primers barcoded-338F (ACTCCTACGGGAGGCAGCA) and barcoded-06R (GGACTACHVGGGTWTCTAAT), targeting a broad diversity of bacteria with few biases against a particular group, were used to amplify the V4 region of the bacterial 16S rRNA gene (Bergmann et al., 2011). The PCRs (50 μL) contained 25 μL Premix Ex Taq (Takara Biotechnology), 13 μL sterilized water, 5 μL of each primer (2 μM), and 2 μL of diluted template DNA (1–10 ng). The PCR conditions were as follows: (i) initial denaturation at 94 ^°C^C for 5 min; (ii) denaturation at 94 ^°C^C for 40 s, annealing at 56 ^°C^C for 60 s, and extension at 72 ^°C^C for 60 s. The second step was repeated 30 times: (iii) elongation at 72 ^°C^C for 10 min. The length and concentration of the PCR products were determined using 1% agarose gel electrophoresis. PCR products were mixed in equal density ratios according to GeneTools Analysis Software (Version 4.03.05.0, SynGene). Then, a mixture of PCR products was purified with an E.Z.N.A. Gel Extraction Kit (Omega, USA) before sequencing on an Illumina Nova6000 platform, and 250 bp paired-end reads were generated (Guangdong Magigene Biotechnology Co., Ltd. Guangzhou, China).

The structure of the rhizobacterial community of *P. orientalis* was analyzed under different water and salinity conditions. The effective reads were merged and grouped into operational taxonomic units (OTUs) in UPARSE at a 97% similarity. Fastp (version 0.14.1) was used to control the quality of the raw data. The primers were removed using Cutadapt software to obtain clean paired-end reads. Paired-end clean reads were merged using usearch-fastq_mergepairs (V10), and the spliced sequences were called raw tags. Fastp was used to control the quality of the raw data to obtain clean paired-end tags. Sequences were clustered into operational taxonomic units (OTUs) at a 97% identity threshold using UPARSE. Taxonomic assignment was carried out using OTUs with SILVA (16 S) and Unite (ITS). Bacterial community abundance indices including Chao1 and ACE and diversity indices including Shannon and Simpson were comprehensively analyzed.

### 2.9 Statistical analysis

Statistical analyses were performed using IBM SPSS Statistics 26 (IBM Corp., USA). Three-way analyses of variance were performed to analyze the effects of time, drought, salinity, and their interactions on xylem water potential. Two-way analyses of variance were performed to analyze the effects of drought, salinity, and their interactions on phytohormone content, leaf chlorophyll content, NSC content, xylem hydraulic and anatomical traits, as well as soil nutrient (N, P, K) content and alpha diversity of rhizobacterial community. The differences between mean values were compared by Duncan’s tests at a significance level of *P*< 0.05. The software Origin 2023 (OriginLab Corp., USA) was used for generating graphs.

## 3 Results

### 3.1 Plant hormone content

**Figure 1:**
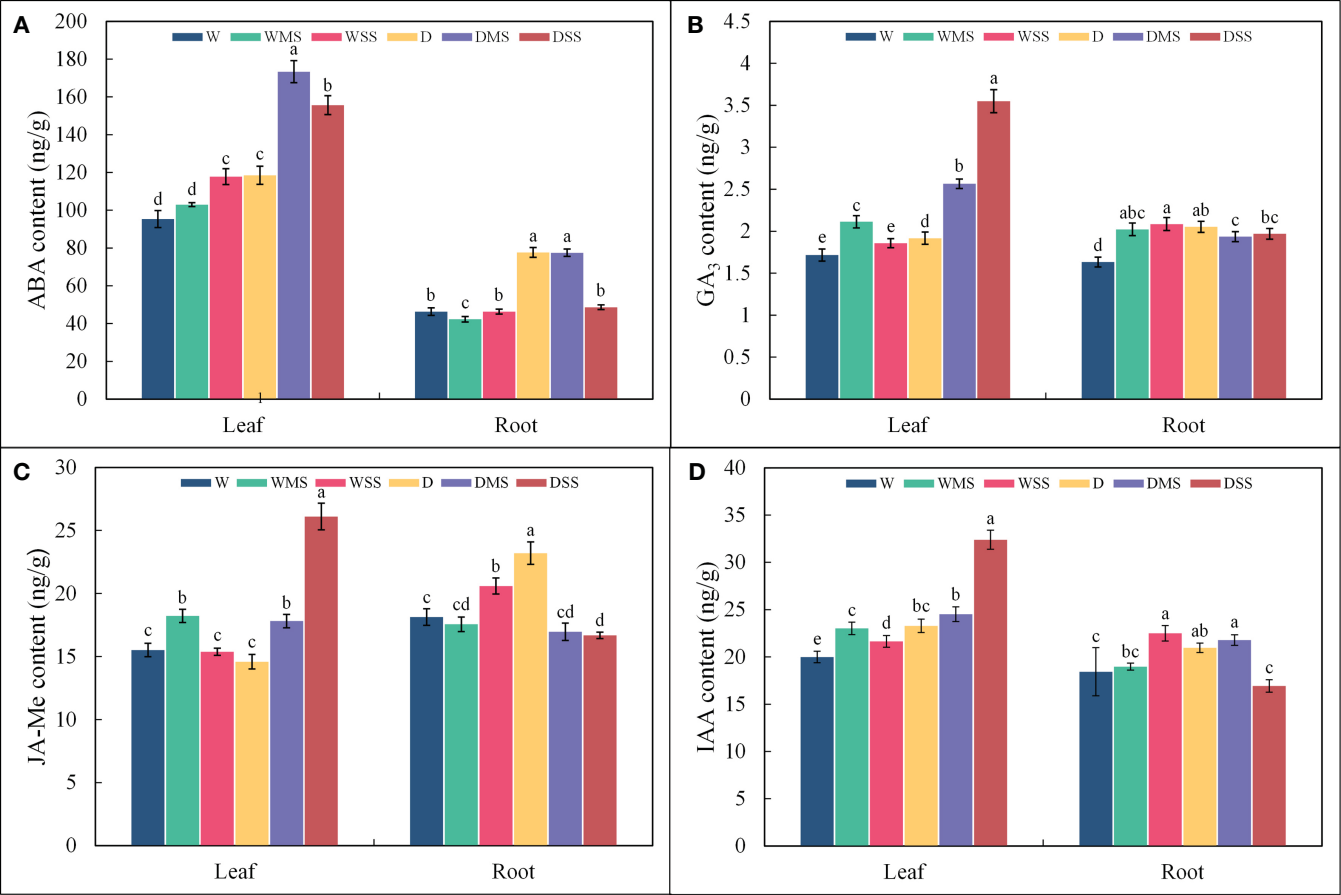
ABA, GA_3_, JA-Me and IAA contents in roots and leaves of *P. orientalis* from different groups. **(A)** ABA; **(B)** GA_3_; **(C)** JA-Me; **(D)** IAA, where different lower-case letters represent significant differences between groups. The phytohormone contents were calculated from at least three samples in each group.

The ABA contents in leaves increased significantly (*P<* 0.05; **Figure 1A**) in the WSS, D, DMS and DSS groups, however, did not change significantly in the WMS group. Root ABA content significantly increased (*P<* 0.05) in the D and DMS groups, significantly decreased (*P<* 0.05) in the WMS group, however, did not show significant change in the WSS and DSS groups (*P* > 0.05) compared with the control group. Leaf GA_3_ contents were significantly higher (*P<* 0.05; **Figure 1B**) in the WMS, D, DMS, and DSS groups than in the control group, and increased more under the combined stress than under the single stress. Root GA_3_ content was significantly higher (*P<* 0.05) than the control under both drought and salt as well as combined stress. Leaf JA-Me content was significantly higher (*P<* 0.05; **Figure 1C**) in the WMS, DMS, and DSS groups than in the control group, but was not significantly different (*P* > 0.05) from the control in the WSS and D groups. The JA-Me content in roots was significantly increased (*P<* 0.05) in WSS and D groups and significantly decreased (*P<* 0.05) in DSS group, whereas in WMS and DMS groups, it was not significantly different (*P* > 0.05) from the control group. Leaf IAA content increased significantly (*P<* 0.05; **Figure 1D**) under both drought and salt stress, whereas root growth hormone increased significantly (*P<* 0.05) in WSS, D, and DMS groups, decreased significantly (*P<* 0.05) in DSS group, and did not change significantly (*P<* 0.05) in WMS group. Overall, drought and salt and their interactions had significant effects (*P<* 0.05) on all four hormones in *P. orientalis* leaves, whereas single drought stress had no significant effect on root JA-Me and single drought or salt stress had no significant effect on root IAA (*P* > 0.05; **Table 1**).

**Table 1:** Significance of the effects of the factors and their interactions on ABA, GA_3_, JA-Me and IAA, where the bolded number representing the p-values less than 0.05.

	ABA		GA_3_		JA-Me		IAA	
P-value	Leaf	Root	Leaf	Root	Leaf	Root	Leaf	Root
*P* (Drought)	**<0.05**	**<0.05**	**<0.05**	**<0.05**	**<0.05**	0.5	**<0.05**	0.891
*P* (Salinity)	**<0.05**	**<0.05**	**<0.05**	**<0.05**	**<0.05**	**<0.05**	**<0.05**	0.523
*P* (Drought × Salinity)	**<0.05**	**<0.05**	**<0.05**	**<0.05**	**<0.05**	**<0.05**	**<0.05**	**<0.05**

### 3.2 Leaf chlorophyll content

**Figure 2:**
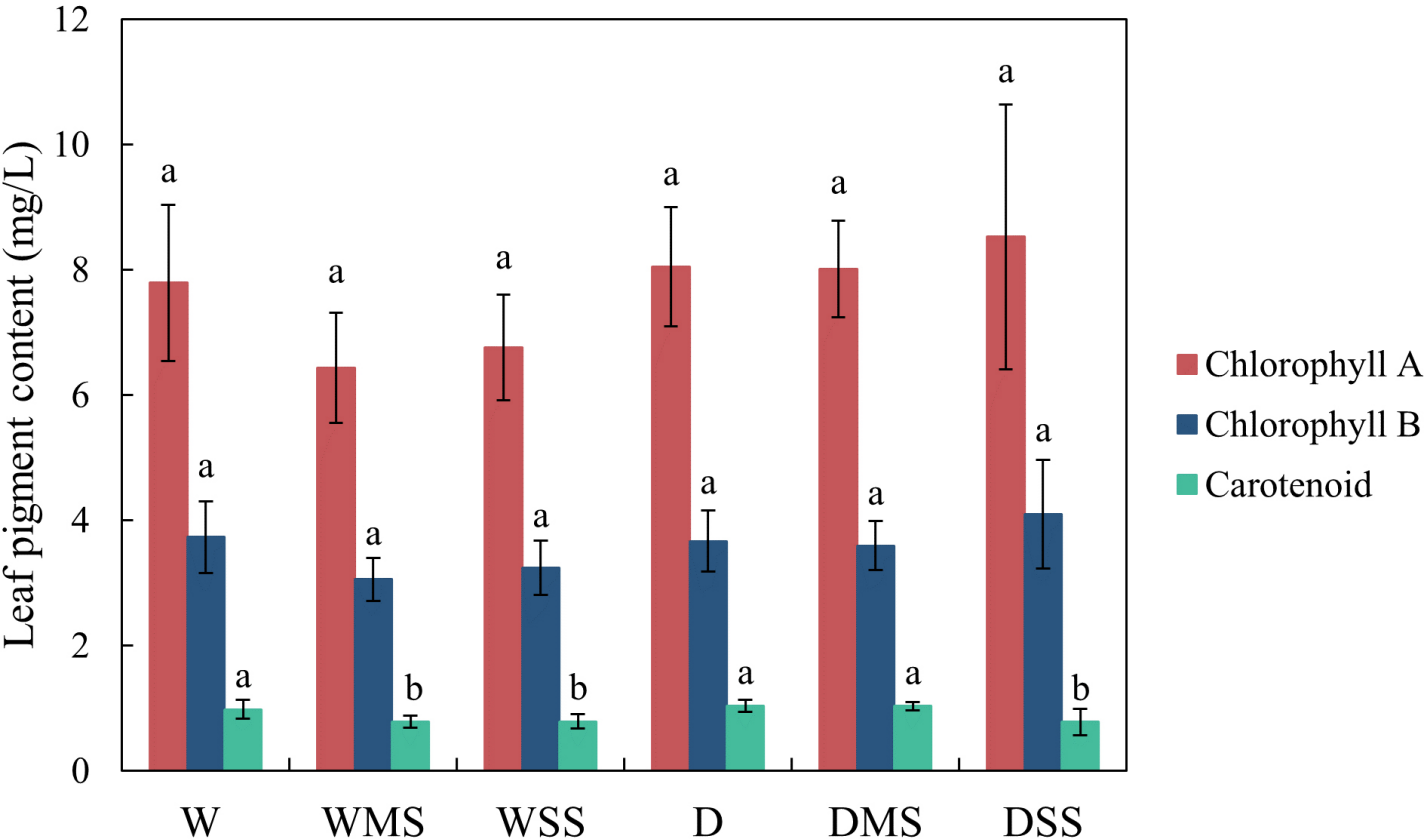
Leaf chlorophyll A, chlorophyll B and carotenoid contents in the different groups. The chlorophyll contents were calculated from at least three samples in each group.

There were no significant changes in chlorophyll A and chlorophyll B contents under drought and salt stress. The carotenoid content was significantly decreased in WMS, WSS, and DSS group (*P* > 0.05; **Figure 2**). In addition, leaf dehydration was visually observed.

### 3.3 NSC content

**Figure 3:**
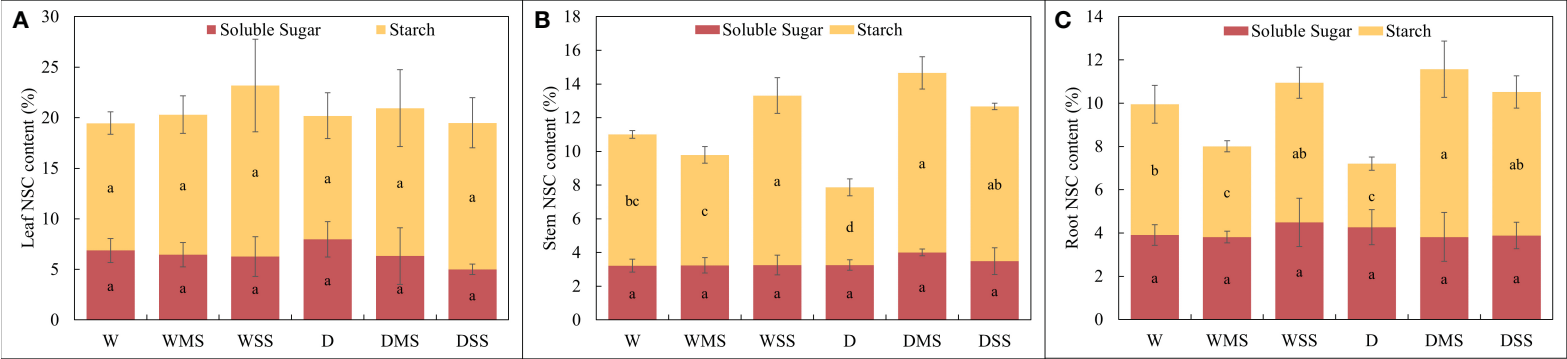
Soluble sugar, starch and NSC concentration in different groups. **(A)** leaf; **(B)** stem; **(C)** root. The soluble sugar, starch and NSC concentrations were calculated from at least three samples in each group.

There were no significant differences in the content of soluble sugars in leaves, stems, and roots among the different groups (*P* > 0.05), and the contents of soluble sugars, starch and NSC in leaves were not significantly different among the groups (*P* > 0.05). However, for stems and roots, the starch concentration significantly decreased in the D group compared with the W group (*P<* 0.05), while the starch and NSC concentration in the DMS and DSS group are significantly higher than the D group (*P<* 0.05). In addition, the results of the interaction analysis showed that both the starch and NSC content of roots and stems were significantly affected by salinity and drought × salinity interaction (**Figure 3**, **Table 2**).

**Table 2:** Significance of the effects of the factors and their interactions on soluble sugar, starch, and NSC, where the bolded number representing the p-values less than 0.05.

	Soluble sugar			Starch			NSC		
P-value	Leaf	Stem	Root	Leaf	Stem	Root	Leaf	Stem	Root
*P* (Drought)	0.894	0.135	0.828	0.610	0.986	0.722	0.640	0.816	0.855
*P* (Salinity)	0.170	0.385	0.714	0.119	**<0.05**	**<0.05**	0.737	0.067	0.059
*P* (Drought × Salinity)	0.441	0.413	0.542	0.616	**<0.05**	**<0.05**	0.457	**<0.05**	**<0.05**

### 3.4 Xylem hydraulic and anatomical traits

**Figure 4:**
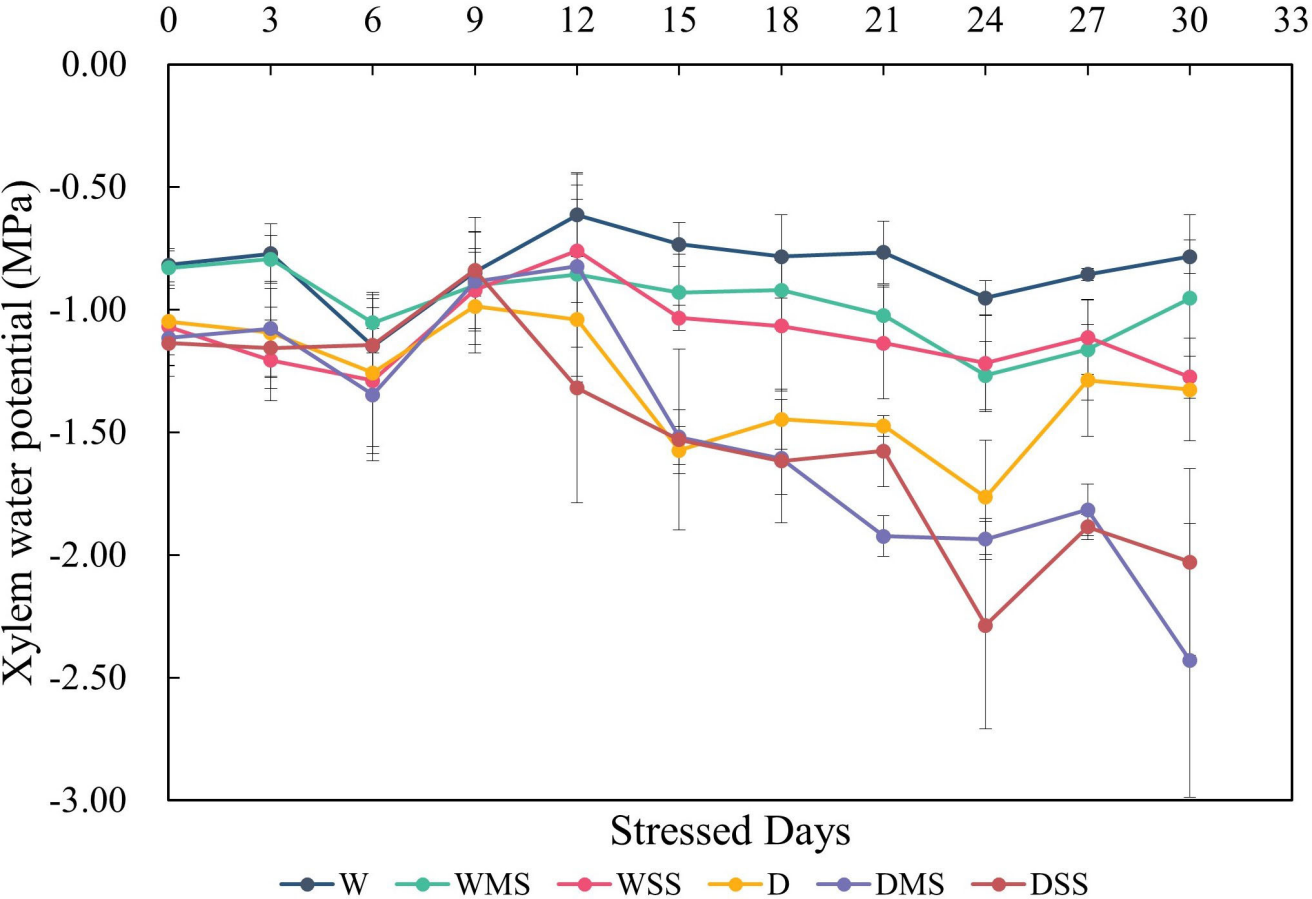
Xylem water potential variations in different groups of *P. orientalis* saplings during stresses. The water potential was calculated from three samples in each group.

**Figure 5:**
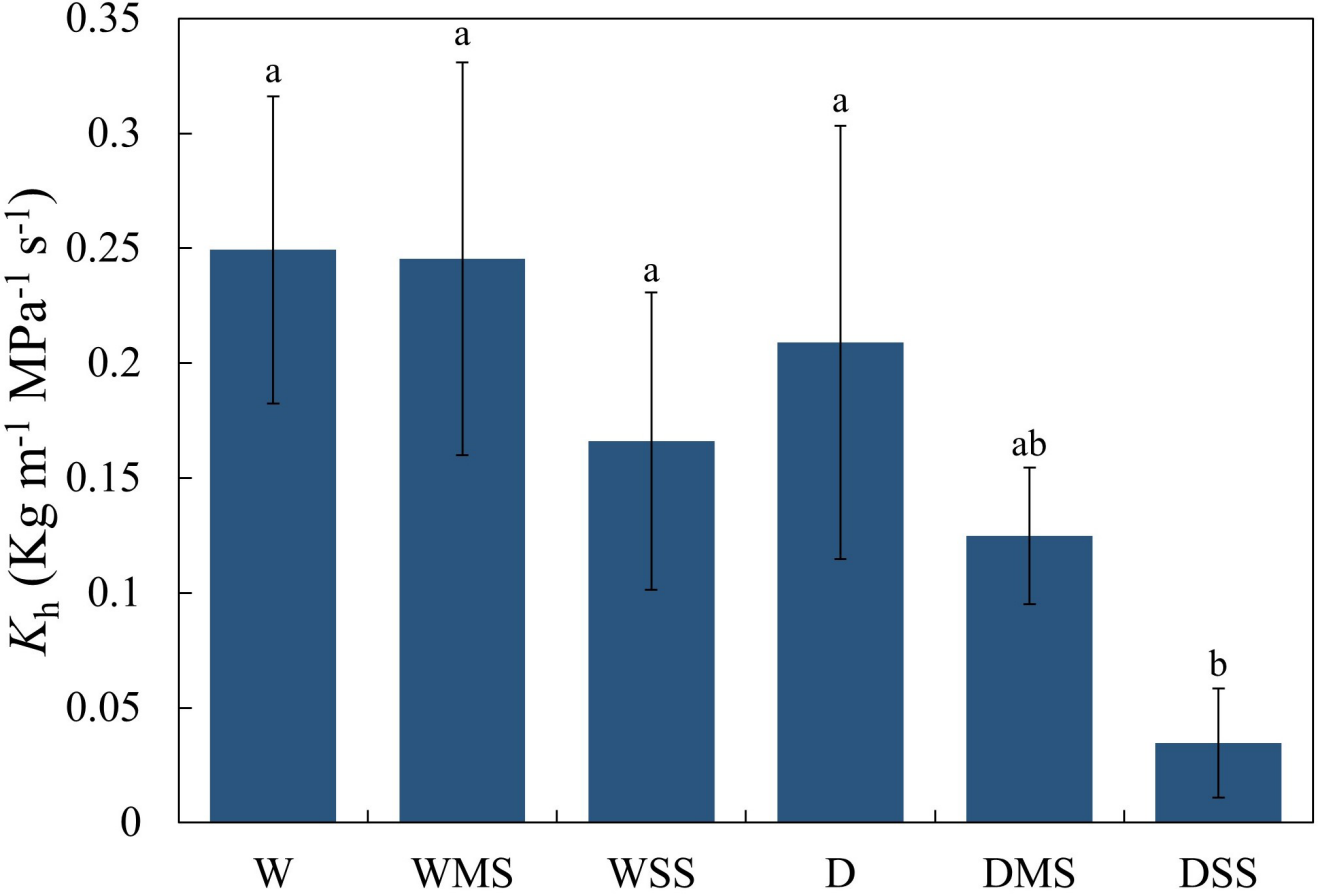
Xylem hydraulic conductivity of different groups. Different lowercase letters indicate significant differences (*P*< 0.05), and the same lowercase letters indicate no significant differences (*P* > 0.05). The xylem hydraulic conductivity was calculated from at least three samples in each group.

Neither drought nor salt stress significantly affected tracheid lumen diameter and tracheid double-wall thickness in roots and stems (*P* > 0.05; **Table 3**). However, the interaction between drought and salt resulted in a significant increase (*P*< 0.05; **Table 3**) in stem tracheid lumen diameter. Under drought and salt stresses, midday leaf water potential of *P. orientalis* saplings decreased significantly with stress duration (*P*< 0.01; **Figure 4**, **Table 4**). There is a significant interaction between drought and salinity with time, drought and salt lead to a progressive decrease in water potential over time, which became statistically significant after 15 days of stress. Therefore, from the 15th day of stress, the water potentials of the D, DMS, and DSS groups were significantly lower (*P*< 0.05) than those of the W, WMS, and WSS groups. There was a negative and significant correlation between xylem water potential and leaf ABA content at the time of sampling (day 30 of stress) (**Figure 1**, **Figure 4**, *P*< 0.05). There was no statistically significant difference between the xylem hydraulic conductivity of the control group and W, WMS, and WSS groups (*P* > 0.05). The mean xylem hydraulic conductivity decreased under the combined stress of drought and salt but was only significantly different between the D and DSS group (*P*< 0.05; **Figure 5**). The results of the interaction analysis showed that both drought and salt significantly affected the xylem hydraulic conductivity of *P. orientalis* saplings, however, there is no interaction between drought and salinity.

**Table 3:** Xylem anatomical traits of different groups of *P. orientalis* saplings and significance of the effects of the factors and their interactions on anatomical traits.

	Tracheid lumen diameter		Tracheid double-wall thickness	
Group	Stem	Root	Stem	Root
W	5.82 ± 1.64ab	6.20 ± 1.51a	3.63 ± 0.29a	
WMS	5.64 ± 1.51ab	6.25 ± 1.47a	3.32 ± 0.53a	3.43 ± 0.61a
WSS	5.09 ± 1.46b	6.13 ± 1.68a	3.62 ± 0.80a	3.96 ± 0.40a
D	5.04 ± 1.58b	5.46 ± 1.93a	3.27 ± 0.71a	3.45 ± 0.62a
DMS	6.31 ± 2.24ab	5.96 ± 1.71a	3.65 ± 0.70a	3.21 ± 0.73a
DSS	7.24 ± 2.27a	6.32 ± 1.83a	3.35 ± 0.52a	3.03 ± 0.45a
*P* (Drought)	0.124	0.715	0.502	0.153
*P* (Salinity)	0.329	0.992	0.709	0.86
*P* (Drought × Salinity)	**<0.05**	0.559	0.636	0.286

Different lowercase letters (a, b, and c) indicate significant differences (P < 0.05), and the same lowercase letters indicate no significant differences (P > 0.05), and the bolded number representing the p-values less than 0.05. The tracheid lumen diameter and tracheid double-wall thickness was calculated from at least three samples in each group.

**Table 4:** Significance of the effects of the factors and their interactions on xylem water potential and hydraulic conductivity, where the bolded number representing the p-values less than 0.05.

Factors	Drought	Salinity	Time	Drought × Salinity	Drought × Time	Salinity × Time	Drought × Salinity × Time
*P* (water potential)	**<0.05**	**<0.05**	**<0.05**	0.170	**<0.05**	**<0.05**	**<0.05**
*P* (hydraulic conductivity)	**0.002**	**0.005**	—	0.414	—	—	—

### 3.5 Soil nutrient (N, P, K) content

Soil nitrate nitrogen contents significantly decreased in the WSS, DMS, and DSS group (*P*< 0.05; **Table 5**). The results of the interaction analysis showed that drought had no significant effect on soil nitrate nitrogen content, while salinity and the interaction of drought × salinity had significant effects on nitrate nitrogen content (*P*< 0.05; **Table 5**). Ammonium nitrogen content was significantly increased in the WMS and DMS group; however, ammonium nitrogen in the D, WSS, and DSS group showed no significant difference from control group (**Table 5**). The results of the interaction analysis showed that only salt stress significantly affected soil ammonium nitrogen (*P*< 0.05; **Table 5**). Drought stress, salt stress, and the combination of the two did not significantly affect soil available phosphorus (*P* > 0.05; **Table 5**). Soil available potassium content increased significantly under single stresses of drought or salt (*P*< 0.05; **Table 5**) but decreased significantly in the DSS group (*P*< 0.05; **Table 5**). Besides, the soil available potassium content followed the pattern that WSS > WMS > W and D > DMS > DSS. The results of the interaction analysis showed that drought did not significantly affect soil available potassium content, while salinity and drought × salinity interaction significantly affected soil available potassium content (**Table 5**).

**Table 5:** Soil nutrient content of different groups and significance of the effects of the factors and their interactions on soil nutrient content.

Group	NO_3_-N	NH_4_-N	AP	AK
W	1006.2 ± 70.1b	65.6 ± 7.5b	422.3 ± 15.6a	2689.6 ± 169.4c
WMS	993.0 ± 59.9b	96.4 ± 2.0a	415.6 ± 40.3a	2937.083 ± 145.5bc
WSS	866.7 ± 45.1c	66.9 ± 8.2b	436.9 ± 2.7a	3187.917 ± 113.1b
D	1122.1 ± 29.5a	58.4 ± 3.7b	503.6 ± 20.2a	4117.083 ± 110.0a
DMS	851.0 ± 25.0c	91.2 ±0.7a	449.3 ± 23.9a	2839.167 ± 172.8c
DSS	869.2 ± 34.5c	80.7 ± 5.4ab	439.5 ± 60.4a	2191.667 ± 89.98d
*P* (Drought)	0.706	0.525	0.114	0.486
*P* (Salinity)	**<0.05**	**<0.05**	0.665	**<0.05**
*P* (Drought × Salinity)	**<0.05**	0.341	0.443	**<0.05**

Different lowercase letters (a, b, and c) indicate significant differences (P < 0.05), the same lowercase letters indicate no significant differences (P > 0.05), and the bolded number representing the p-values less than 0.05. The soil nutrient content was calculated from three samples in each group.

### 3.6 Rhizobacteria community

**Figure 6:**
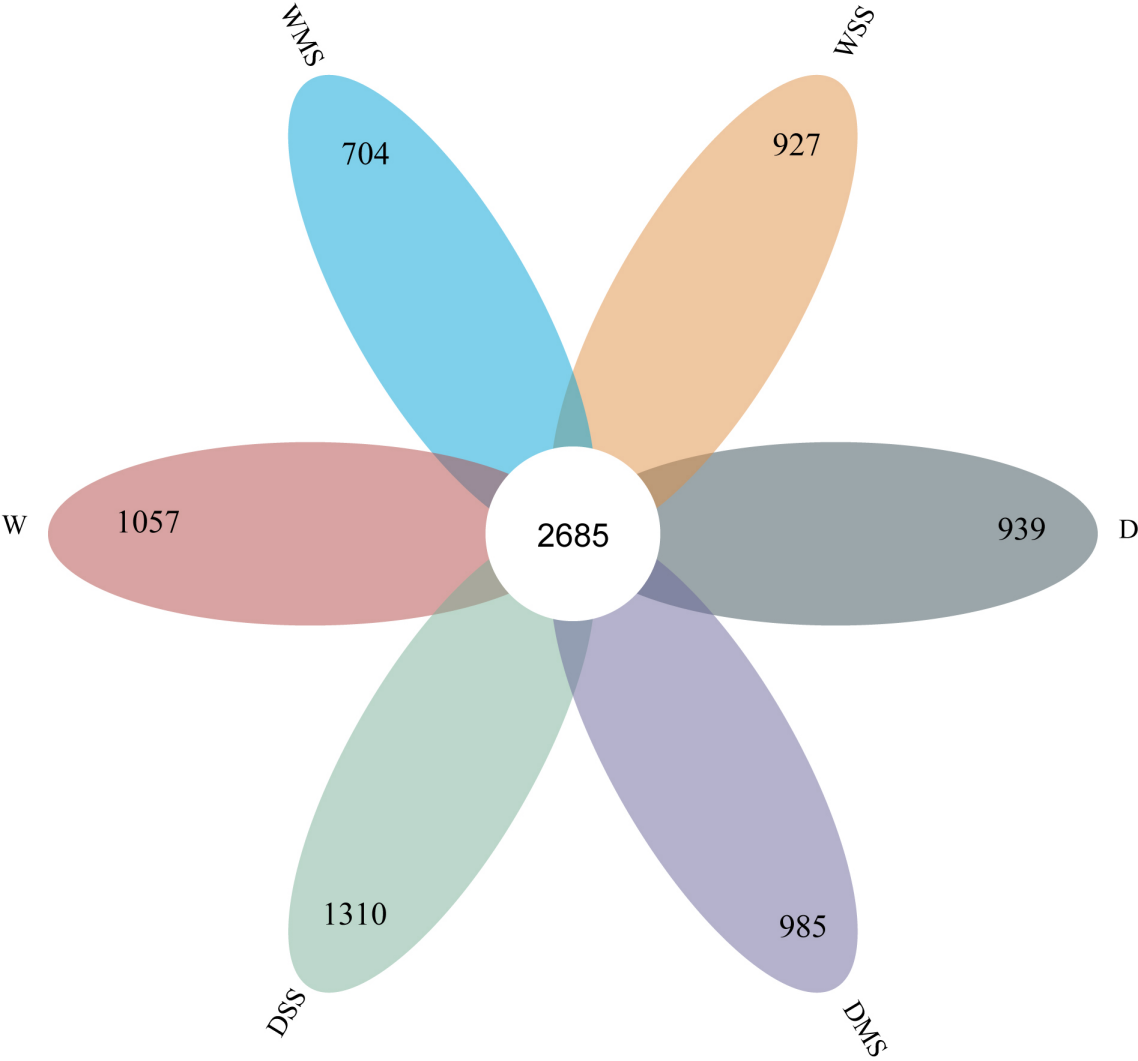
Venn diagram of rhizobacteria OTU in different groups.

**Figure 7:**
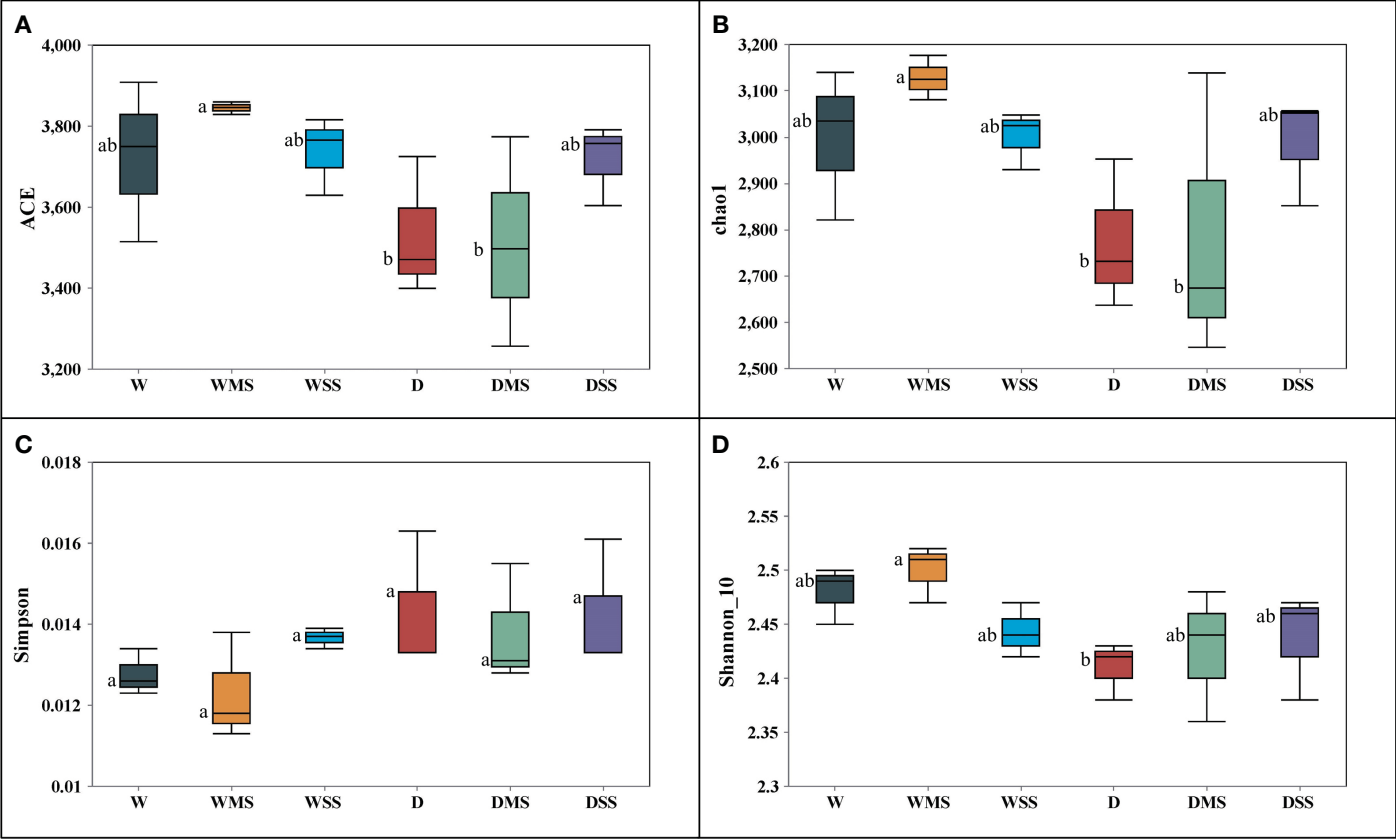
Indices reflecting alpha diversity of rhizobacteria among different groups. **(A)** ACE index; **(B)** chao1 index; **(C)** Simpson index; **(D)** Shannon_10. The alpha diversity was calculated from three samples in each group.

In total, 2,685 OTUs were present in the different groups, and the number of OTUs unique to the different groups ranged from 704 to 1,310 (**Figure 6**). For the 18 samples in the six groups, the coverage index for each replicate reached 0.98 or more, indicating that most of the bacterial sequences could be covered by sequencing. Drought stress significantly affected the alpha diversity of rhizobacteria community. Specifically, single drought stress reduced ACE, chao1, and Shannon indices, as well as increased Simpson index, suggesting a decrease in rhizobacterial abundance and diversity. Nevertheless, single salt stress and the combined stress of drought and salinity did not significantly affect rhizobacterial alpha diversity (*P* > 0.05; **Table 6**; **Figure 7**).

**Table 6:** Significance of the effects of the factors and their interactions on abundance and diversity of rhizobacteria community, where the bolded number representing the p-values less than 0.05.

Factors	Drought	Salinity	Drought × Salinity
*P* (ACE)	**<0.05**	0.580	0.271
*P* (chao1)	**<0.05**	0.548	0.271
*P* (Simpson)	0.071	0.500	0.766
*P* (Shannon_10)	**<0.05**	0.559	0.278

## 4 Discussion

### 4.1 Effects of single and combined stress of drought and salinity on the biochemical traits

In our study, compared with the control group, leaf ABA content in *P. orientalis* increased significantly under drought and salt stress. ABA induces stomatal closure to decrease water loss and ion transport from root to shoot (Li et al., 2023). Therefore, the increase of ABA is one of the responses to drought and salt stress in *P. orientalis* saplings. Single drought stress and combined stress of drought and moderate salt stress resulted in significantly higher root ABA content, while root ABA content under combined stress of drought and severe salt stress was not significantly different from the control. Previous studies have shown that Cupressaceae tree species exhibit p-type ABA dynamics under severe drought, i.e., ABA levels increase early in the drought and decrease as the water potential further decreases (Brodribb et al., 2014; Mercado-Reyes et al., 2023). Similar reasons may explain the lower root ABA in DSS group compared with DMS group in our study. Roots are the primary site of ABA synthesis, and the decrease in root ABA content may result from inhibited synthesis under stress (Mercado-Reyes et al., 2023). We observed significantly lower leaf ABA in the DSS group than in the DMS group and hypothesized that p-type ABA dynamics also applied in the leaves of *P. orientalis* saplings.

The gibberellin content of both roots and leaves increased under drought and salt stress. Gibberellins have positive effects on plant growth and tolerance under drought and salt stress (Moula et al., 2020; Gaion and Carvalho, 2021). JA and JA-Me are endogenous growth regulators, exhibit phytohormones crosstalk with ABA in the pathway for stomatal closure (Xing et al., 2022), and can also improve plant tolerance to drought and salt (Seo et al., 2005; Daszkowska-Golec and Szarejko, 2013). Under abiotic stresses such as drought and salinity, the JA signaling factor *PeJAZ2* in *Populus euphratica* interacts with *PeOST1*, which is involved in stomatal regulation. The overexpression of *PeJAZ2* activates the expression of ABA signal transduction genes, leading to the induction of stomatal closure and an increase in water use efficiency (Rao et al., 2023). The significant increase in leaf JA-Me content under the stresses may improve the tolerance of *P. orientalis* to salt stress in the form of induced stomatal closure. However, the role of JA in plant salt tolerance mechanism varies among species. For instance, both exogenous JA-Me and the accumulation of endogenous JA enhance the salt tolerance of *Abelmoschus esculentus* (Wang et al., 2023). Conversely, in *Oryza sativa*, the accumulation of endogenous JA and its signal transduction negatively affected salt tolerance (Ndecky et al., 2023). In this study, there was no significant increase in roots in JA-Me in the DMS and DSS group. We hypothesize that the ability of *P. orientalis* to improve stress tolerance through hormonal regulation is limited, and the combined stress of drought and salt exceeds this limitation, leading to dysfunction starting from the roots. IAA contributes to the development of plant vascular tissues and plays an important role in plant growth and development (Ryu and Cho, 2015), and its content in leaves increased under drought and salt stress. IAA can regulate xylem size (Junghans et al., 2006), and an increase in the IAA content may have contributed to the increase in tubular lumen diameter in the DSS group. There is a synergistic effect between IAA and GAs, with IAA promoting the biosynthesis of GAs (Zhu et al., 2022) and GAs promoting the transport of IAA (Li et al., 2015). In our study, both drought and salt stress resulted in a significant increase in leaf GA_3_ and IAA content, which may be due to the synergistic effect of these two. In addition, exogenous application of IAA with GAs increased superoxide dismutase (SOD) and peroxidase (POD) activities of *Solanum tuberosum* under salt stress and had a positive effect on its growth (Khalid and Aftab, 2020).

Overall, under single stress, the content of ABA, JA-Me and GA_3_ in *P. orientalis* saplings significantly increased, which can enhance the resistance to abiotic stresses (Colebrook et al., 2014; Sah et al., 2016; Wang et al., 2021b). However, under combined stress of drought and severe salt, the content of ABA and JA-Me in roots did not significantly increase, indicating that hormone regulation ability of *P. orientalis* saplings was limited.

Chlorophyll A and B are the main pigments involved in light harvesting and energy transfer in photosynthesis, while carotenoids are accessory pigments that protect the photosynthetic apparatus from excess light and oxidative damage (Sonobe et al., 2020). Drought stress can reduce the chlorophyll content of plants by affecting the biosynthesis and degradation of chlorophyll, while salt stress can also decrease the chlorophyll content of plants by impairing the chloroplast structure and function (Hamani et al., 2020; Song et al., 2021). In addition, the increase of ABA, GAs, JA-Me and IAA may inhibit chlorophyll synthesis or promote its degradation (Liu et al., 2017; Zhu et al., 2017). However, in our study, chlorophyll A and chlorophyll B of *P. orientalis* saplings were not significantly affected by drought, salt, and their combined stresses, which is consistent with Acosta-Motos et al. (2015). In this study, chlorophyll content was expressed as the ratio of chlorophyll extract content to leaf fresh weight. Under drought and salt stress, plant leaves are usually dehydrated (Chaves et al., 2009; Kuromori et al., 2022), which is consistent with our findings. Therefore, we hypothesize that the decrease in fresh weight due to leaf dehydration and the decrease in chlorophyll extract content may occur at the same time, which in turn leads to a lack of significance in the changes in chlorophyll content that we calculated (Hou et al., 2015). In addition, species that are tolerant to both drought and salt may not show significant change on the chlorophyll content (Anjum et al., 2011; Acosta-Motos et al., 2017), and *P. orientalis* is tolerant to both drought and salt (Ou et al., 2023), therefore its chlorophyll content didn’t change under drought and salt stress, which may help to maintain its photosynthetic capacity. This further leads to unchanged leaf NSC content in our study.

Under single drought stress, the NSC content of stem and root was decreased, which was in agreement with other studies (He et al., 2020; Ji et al., 2020). Besides, the NSC content of different organs responded differently to drought and salt stress. Leaf NSC contents did not change significantly in any group, while the NSC contents in stems and roots were significantly increased in the DMS group and significantly decreased in the D group, indicating that the effects of combined stress on NSC content are not additive. The NSC content of root and stem was significantly higher in the DSS and DMS group than the D group, however, the pattern does not apply to the WSS, WMS and W group (**Figure 3**). This suggested that the effect of salinity on NSC content varied depending on soil moisture content, i.e., there was an interaction between drought and salt stress, which was positive on the NSC content in our study. NSC content significantly decreased in roots under single drought stress but increased or remained unchanged under combined stress, indicating that the effects of combined stress on NSC content are not additive. Previous studies have shown that salt addition can mitigate the negative effects of drought on NSC content in *Robinia pseudoacacia*, which is consistent with our results (Fan et al., 2024). Likewise, salt addition increased water use efficiency and protected dry matter production of *Atriplex lentiformis* under drought stress (Glenn et al., 2012). In our study, root NSC content significantly increased in the DMS group, reflecting the strong carbon storage capacity of *P. orientalis* under combined stress, as well as the positive interaction between drought and salt stress on NSC content. However, the interaction between drought and salt is complex and its effects may be species-specific. For example, under drought, salt and their combined stress, the starch and NSC content of *Juglans regia* L. and *Tamarix chinensis* was significantly decreased (Wang et al., 2021a; Mao et al., 2023), while the NSC content in the roots of *Triticum aestivum under* combined drought and salt stress was significantly increased (Fu et al., 2023).

In summary, our results suggest that the NSC contents of roots and stems in *P. orientalis* are more responsive to salt stress than leaves. Also, starch content is more sensitive to the stresses than soluble sugar content. Fluctuations in starch and NSC contents reflect the osmotic adjustment ability under drought and salt stresses, while stable soluble sugar levels suggest *P. orientalis* can maintain carbon supply ability under stresses (Thalmann et al., 2016). Further studies are needed to elucidate the molecular mechanisms of these metabolic changes to clarify the different patterns of changes in NSC content under different stresses.

### 4.2 Effects of single and combined stress of drought and salinity on the xylem anatomical and hydraulic traits

Our study showed that drought and salt stress affected the lumen diameter of xylem tracheids in *P. orientalis*. Under well-watered conditions, the lumen diameter decreased with increasing salt concentration, although it is insignificant, which is consistent with Rajput et al. (2015), indicating that salt stress slightly reduced the water transport capacity of the stem xylem. This could be due to the osmotic stress and ion toxicity caused by salt stress, which may impair the expansion of xylem tracheids (Li et al., 2023). Surprisingly, tracheid lumen diameter of stems increased under combined drought and salt stress, which may be related to the significant increase in IAA content (Junghans et al., 2006). The variation in vessel diameter of poplar conduits under salt stress were related to IAA content (Ryu and Cho, 2015), although the mechanisms regulating the variation of xylem anatomical traits under salt stress are still unknown (Eckert et al., 2019). However, no significant changes in root and stem tracheid double-wall thickness were observed under drought and salt stresses. Overall, the anatomical structure of *P. orientalis* showed limited changes under drought and salt stress, and the combined stress of drought and salt on tracheid diameter resulted in effect that is different from any single stress.

Drought and salt stress could result in reduced xylem water potential and hydraulic conductivity (Li et al., 2023). The results of this study also show that drought and salt stresses affect the xylem water potential. The decrease in leaf water potential was more pronounced in the D, DMS, DSS group than in the W, WMS, WSS group, suggesting that drought stress had a stronger effect on xylem hydraulic traits than salt stress. ABA is an important phytohormone for the regulation of xylem water potential under drought and salt stress (Zhang et al., 2006). In our study, the negative and significant relationship between ABA content and xylem water potential confirmed the regulation of ABA on xylem water potential in *P. orientalis* saplings (**Figure 1**, **Figure 4**). The xylem hydraulic conductivity did not change significantly in the WMS and WSS group, suggesting that the *P. orientalis* saplings were able to maintain their water transport capacity under single salt stress. The xylem hydraulic conductivity in the D group also showed no significant difference from the W group. This may be due to the mild degree of drought in this study, which did not induce xylem embolism in *P. orientalis* saplings. However, although the xylem tracheid lumen diameter increased in the DSS group, the xylem hydraulic conductivity decreased significantly in the DSS group, which may be related to the occurrence of xylem embolism and the inhibition of aquaporins activity (Shekoofa and Sinclair, 2018). In addition, a lower ratio of GA_3_/ABA was found to inhibit leaf growth and reduce water loss (Zhu, 2023). In our study, the ratio of GA_3_/ABA was significantly reduced in WSS, D, and DMS groups (*P*< 0.05), which may maintain the xylem hydraulic conductivity. However, the ratio of GA_3_/ABA was significantly increased in the DSS group (*P*< 0.05), which may have increased water loss, leading to a significant decrease in xylem hydraulic conductivity and demonstrated the limit of hormone regulation in *P. orientalis* seedlings. Overall, combined stress leads to a more severe loss of xylem hydraulic conductivity compared with single stress.

Previous studies have shown that both single or combined stress of drought and salt can lead to a decrease in hydraulic conductivity and NSC content (Wang et al., 2021a; Cao et al., 2023), resulting in hydraulic failure and carbon starvation, which is recognized as the main mechanisms of tree mortality (McDowell, 2011; McDowell et al., 2022). In our study, NSC content in stems and roots was significantly decreased under single drought stress, whereas the NSC content of all organs was not decreased under the combined stress, suggesting that combined stress of drought and moderate salt can offset the negative effects of single drought stress on NSC content in *P. orientalis* saplings. However, when drought was combined with severe salt stress, there was no significant change in NSC content, while the hydraulic conductivity had been reduced by approximately 86%. Therefore, when drought is combined with severe salinity, the risk of hydraulic failure occurs much earlier than carbon starvation under combined stress, and further research is needed on investigating the mechanisms of tree mortality under combined stress.

### 4.3 Effects of single and combined stress of drought and salinity on the soil properties

The results of this study showed that drought and salinity have different effects on soil nitrogen and phosphorus availability. Nitrogen and phosphorus are essential nutrients for plant growth and development, and their uptake and metabolism are influenced by soil water and salt conditions. Salt stress may inhibit the decomposition of soil organic matter to reduce potential mineralized N, as well as inhibit nitrification and denitrification by soil microorganisms, and ultimately significantly affect soil nitrate and ammonium N levels. Drought stress may not affect soil nitrate and ammonium nitrogen contents significantly, but it may reduce the plant uptake and translocation of these nutrients due to the low soil water potential (Tian et al., 2021). Soil available phosphorus comes mainly from organic matter decomposition and mineral dissolution. Phosphorus is one of the most immobile nutrients in soils and increased soil salinity reduces the rate of phosphorus uptake by plants, so soil available phosphorus levels may not change significantly under drought and salt stress (Miura, 2012; Su et al., 2021). These findings imply that salinity stress has a greater impact on soil nitrogen cycling than drought stress, and that nitrogen management is more critical than phosphorus management under saline and dry conditions. In our study, drought led to an increase in soil available potassium content, which is consistent with Shi et al. (2010). Plants can only take up potassium from the soil solution (Ashley et al., 2005); therefore, under drought stress, the uptake of potassium by plants decreases (Wang et al., 2018), resulting in higher potassium content remaining in the soil. The decrease in soil available potassium due to the combined drought and salt stress may be due to the competition between Na and K in the soil for the exchange places (Wang et al., 2013; Stavi et al., 2021), resulting in a decrease in K availability. However, soil effective potassium content increased under salt stress, probably due to salt-induced K efflux and reduced K uptake in plants (Wu et al., 2018). Considering that our study results were based on the pot experiment, further investigations could be done to examine the response of natural soil nutrient content under the combined stresses of drought and salt.

Physiological changes in plants under drought or salt stress, such as changes in root exudates, affect the rhizobacteria community (Balestrini et al., 2024). Our results showed that drought and salt stress have different effects on the rhizobacteria alpha diversity. Soil bacterial abundance and diversity are important indicators of soil health and function, and they are influenced by various environmental factors, such as soil pH, moisture, nutrients, and salinity (Lelana et al., 2022; Li et al., 2022a). In this study, drought significantly decreased the ACE, Chao1 and Simpson indices (**Table 6**), indicating that drought reduced the abundance and diversity of rhizobacteria community. This could be due to the water loss, nutrient variation, and osmotic imbalance caused by drought stress (Kim et al., 2017). However, salt stress did not significantly affect all the indices shown, indicating that soil bacterial abundance and diversity was not affected by salinity. Similar to the our study, salinity did not significantly affect the alpha diversity of rhizosphere microbial community of *Spinacia oleracea* (Ibekwe et al., 2017). Our results suggest that drought stress is more detrimental to alpha diversity of rhizobacteria community than salt stress, and that there is no interaction between drought and salinity. In addition, the use of commercial nutrient soils may result in soil microbial responses that differ from the natural soil. More studies in natural soil are needed to further elucidate the mechanisms by which drought and salinity affect rhizosphere microbial communities.

## 5 Conclusions

Our study showed that single drought and salt stress and their combined stress affected various physiological traits in *P. orientalis* saplings. The responses of *P. orientalis* saplings under single and combined stress of drought and salt were diverse, and effects of combined stress could not be directly extrapolated from any single stress. ABA, GA_3_, JA-Me and IAA contents were significantly increased in leaves and roots under most stress conditions. Drought stress reduced the starch storage in stems and roots, while the combined stress of drought and salt stress did not decrease it. The combined stress of drought and salinity resulted in higher starch and NSC contents in stems and roots than single drought stress. The content of soluble sugars in different organs was not affected by any stress. NSC content significantly decreased in roots under single drought stress but increased or remained unchanged under combined stress, indicating that the effects of combined stress on NSC content are not additive. Xylem water potential of *P. orientalis* saplings were significantly reduced under both drought and salt stress. Xylem hydraulic conductivity was only significantly decreased under the combined stress, while NSC content was unaffected, demonstrating that the risk of xylem hydraulic failure due to combined stress is greater than carbon starvation. The anatomical structure of root and stem xylem was hardly affected, except for the stem xylem tracheid lumen diameter which was significantly increased under the combined stress of drought and severe salt. Drought stress affected rhizobacteria alpha diversity, however, under any of the stresses, the alpha diversity of rhizobacteria communities was not significantly different from the control. Salt stress significantly reduced nitrate nitrogen content and increased ammonium nitrogen content. Available phosphorus content did not change significantly, and soil available potassium was significantly affected by salinity. Overall, salt stress has a greater impact on soil nutrient availability, whereas drought stress more significantly affects the alpha diversity of rhizobacteria communities, and the interaction of drought and salt has limited effects on soil properties. Regarding physiological traits, responses of *P. orientalis* saplings under single and combined stress of drought and salt exhibited variability, indicating that the effects of combined stress cannot be directly extrapolated from any single stress. Notably, compared to single stress, the effect of combined stress on phytohormone content and xylem hydraulic traits was negative to *P. orientalis* saplings, while the negative effects of single drought stress on NSC content were offset under the combined stress. Our results suggest that *P. orientalis* may have different strategies to cope with drought and salinity stress, however, the mechanisms leading to the different responses to drought and salt as well as combined stresses remain unclear. An intensive study of the response mechanisms of this species to single and combined stress of drought and salt is essential to improve our understanding of it. Therefore, it is necessary to investigate the similarities, differences, and interactions between the molecular mechanisms of drought and salt-induced plant responses in future studies.

## Data availability statement

The datasets presented in this study can be found in online repositories. The names of the repository/repositories and accession number(s) can be found below: NCBI SRA, SRR27143335-SRR27143352.

## Author contributions

ShL: Conceptualization, Funding acquisition, Methodology, Project administration, Resources, Supervision, Writing – review & editing. SeL: Data curation, Formal analysis, Investigation, Methodology, Validation, Visualization, Writing – original draft. JW: Investigation, Writing – review & editing. ZL: Investigation, Writing – review & editing. CY: Investigation, Writing – review & editing. MW: Investigation, Writing – review & editing. JG: Resources, Supervision, Writing – review & editing.
